# The Medium- to Long-Term Results of Vascular-Sparing Subcapital Osteotomy (VASSCO) for Pediatric Patients with Chronic Slipped Capital Femoral Epiphysis

**DOI:** 10.3390/jcm13041021

**Published:** 2024-02-10

**Authors:** Cesare Faldini, Alberto Di Martino, Matteo Brunello, Niccolò Stefanini, Nicole Puteo, Federico Pilla, Giuseppe Geraci, Francesco Traina

**Affiliations:** 1 Orthopaedic Department, IRCCS—Istituto Ortopedico Rizzoli, via Giulio Cesare Pupilli, 1, 40136 Bologna, Italy; cesare.faldini@ior.it (C.F.); matteo.brunello@ior.it (M.B.); niccolo.stefanini@ior.it (N.S.); federico.pilla@ior.it (F.P.); giuseppe.geraci@ior.it (G.G.); francesco.traina@ior.it (F.T.); 2 Department of Biomedical and Neuromotor Science—DIBINEM, University of Bologna, 40126 Bologna, Italy; nicole.puteo@ior.it; 3 Ortopedia, Traumatologia e Chirurgia Protesica e dei Reimpianti di Anca e Ginocchio, IRCCS Istituto Ortopedico Rizzoli, 40136 Bologna, Italy

**Keywords:** slipped capital femoral epiphysis, osteotomy, surgical technique, avascular necrosis, tips, outcomes

## Abstract

**Background:** In patients affected by chronic slipped capital femoral epiphysis (C-SCFE), the performance of a subcapital osteotomy is an effective procedure to correct the deformity at the proximal femur. However, the rate of postoperative complications is very high, with iatrogenic avascular necrosis of the femoral head (AVN) being the most bothersome. To overcome the risk of AVN, the modified Dunn procedure according to Ganz and, more recently, the Vascular Sparing Subcapital Osteotomy (VASSCO) technique have been proposed; however, only short-term follow-up studies are available on the latter technique being used. The aim of this study is therefore to show our mid-term clinical and radiological results with the VASSCO technique. **Materials and Methods:** A total of 26 patients underwent VASSCO for moderate or severe stable C-SCFE between 2012 and April 2016 with an average 10-year follow-up (range 8–12 years). The outcomes was evaluated using the HHS and pre- and postoperative ROM. The radiological outcomes and complications were collected. **Results**: No major intraoperative complications occurred; three patients reported postoperative transient apraxia of the lateral femoral cutaneous nerve, which completely recovered in six months. All the radiological outcomes showed substantial improvement postoperatively. One case patient developed AVN of the femoral head and required a total hip arthroplasty after 12 years. **Conclusions:** The current data suggest that VASSCO osteotomy is a reliable technique with very good clinical results at mid-term follow-up; it could be considered a valuable alternative to using more complex techniques to restore the proximal femoral anatomy in moderate to severe C-SCFE.

## 1. Introduction

Chronic slipped capital femoral epiphysis (C-SCFE) is a developmental deformity of the proximal femur responsible for lower limb shortening and femoral neck angulation with CAM impingement, ultimately leading to secondary hip degenerative arthritis [1,2,3,4]. Its incidence is underestimated, especially in the mild forms; in Italy, it occurs in 2.9 cases/100,000 inhabitants, while in the US, it reaches 10.8/100,000 children [5]. To manage the deformity associated with C-SCFE, in 1964, Dunn [6] proposed a technique of subcapital osteotomy using a posterior approach that required a trochanteric osteotomy; he reported an associated risk of avascular necrosis of the femoral head (AVN) of only 4%. The advantage of the performance of a subcapital osteotomy is that the deformity is corrected at the apex, preventing joint degeneration at long-term follow-up [6,7,8]. Two subcapital osteotomy techniques have been proposed thereafter to overcome the risk of AVN, the so-called “modified Dunn procedure”, performed through a hip dislocation according to the technique proposed by Ganz [9,10,11,12,13], and an “in situ” Vascularity Sparing Subcapital Osteotomy (VASSCO), which is performed using an anterior minimally invasive approach to the hip joint [14].

In the literature, necrosis is commonly reported as a complication during these procedures [9,10,11,12,13,15], with the rates ranging from 3% to 24% [16]. The performance of a subcapital osteotomy using a direct anterior approach was first popularized by De Rosa et al. [17] in the late 90s, but he reported a 15% risk of AVN. VASSCO was developed by the authors in 2016, and its preliminary results [14] positively showed no occurrence of AVN. The VASSCO technique appeared effective in sparing the vascular supply of the femoral head; it combined the surgical techniques and tools derived from spine surgery and the anterior minimally invasive approach used for total hip replacement [18,19,20,21,22]. The main advantage of the VASSCO technique was that it did not require hip dislocation, reducing the risk of AVN related to this maneuver, the surgical time, and the overall bleeding. However, the follow-up of the original report was relatively short, averaging 28 months. At present, no study has analyzed the medium- to long-term results when VASSCO is performed in the pediatric population for C-SCFE. The aim of this study was therefore to report the medium–long clinical and radiological results of patients operated on using VASSCO with a follow-up of 10 years.

## 2. Materials and Methods

### 2.1. Study Design and Patients

Between 2012 and 2016, 26 pediatric patients with moderate to severe stable C-SCFE were referred to the authors’ institution and were included in the study. The inclusion criteria were patients being affected by proximal femur deformity due to C-SCFE, VASSCO as the surgical technique, patients being in the growing age, and a follow-up of least 8 years. The exclusion criteria were proximal deformity secondary to other diseases, patients being adults, and those treated using techniques other than VASSCO. The indications for surgical correction of the deformity were stable slips, moderate–severe hip pain and functional compromise, and a shortened and externally rotated lower limb.

### 2.2. Surgical Technique

**Figure 1:**
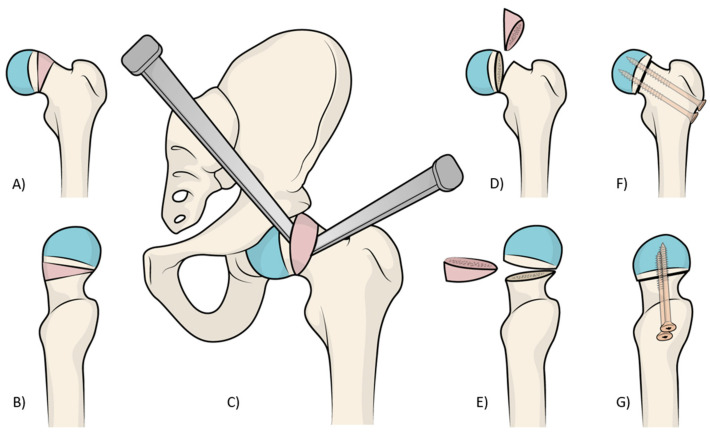
In images (**A**,**B**), it is possible to understand the pathological anatomy of chronic slipped capital femoral epiphysis, with a varus and retroverted neck. In figure (**C**), is shown how, with the help of two osteotomes, a cuneiform wedge resection with an anterolateral base is performed. The wedge osteotomy removes all the prominent pathologic tissue (a bump that is clearly visible at the level of the epiphyseal plate) in the coronal (**D**) and sagittal planes (**E**). The final correction after the reduction, stabilized using two screws in the coronal (**F**) and sagittal planes (**G**).

The surgical procedure (Figure 1) was performed according to the original report [14], usually performed under general anesthesia with the patient placed supine on a traction table commonly used for hip fractures. The hip is placed in a neutral position with a flexion of 20°. The contralateral limb is placed in flexion–abduction on a dedicated leg holder.

Briefly, a modified direct anterior approach to the hip was performed according to Faldini et al. [21], with the skin incision starting one finger distal and lateral from the anterior superior iliac spine and prolonged for 7–8 cm toward Gerdy’s tubercle. Once the lateral circumflex artery is exposed, which usually runs at the distal third of the surgical field beneath the muscular fascia, it should be carefully protected to preserve the blood supply at the femoral head. In the proximal 2 thirds of the surgical field, the reflected head of the rectus femoris covers the medial part of the hip capsule, and it is detached. It is then retracted medially, together with the sartorius and iliopsoas muscles, using a Hohmann retractor, which surrounds the inferior arch of the neck. A second Hohmann retractor is positioned supero-laterally over the capsula of the femoral neck, retracting laterally the tensor fasciae latae and gluteus medius muscles.

A third Hohmann retractor or a Kocher retractor is placed supero-medially to expose the acetabular rim. Before performing a T-shaped capsulotomy, the hip should be placed in mild external rotation to tension the capsule. The horizontal part of the capsular incision is performed 5 mm distal and lateral to the acetabular rim to avoid damage to the acetabular labrum. Once the vertical incision is performed, the two capsular flaps are stitched and retracted, and 2 Hohmann retractors are placed to surround the femoral neck. The leg is then positioned in adduction and external rotation to expose the deformed tissue of the epiphyseal plate at the femoral neck (Figure 1). A cuneiform wedge resection with an anterolateral base is performed using a saw and completed using a straight or curved osteotome. The wedge osteotomy removes all of the prominent pathologic tissue (a bump that is clearly visible at the level of the epiphyseal plate) responsible for the mechanical conflict between the femur and the acetabulum.

The osteotomy should be incomplete to leave a hinge at the postero-medial cortex and begin proximally; the pathologic tissue (Figure 1) is completely removed using rongeurs and curettes to allow a good visualization of the hinge at the opposite cortex. Then, the remaining bone is carefully removed using a Kerrison rongeur, with care not to violate the underneath periosteum. In this fashion, the posterior–superior retinacula containing the vascular branches of artery are preserved.

**Figure 2:**
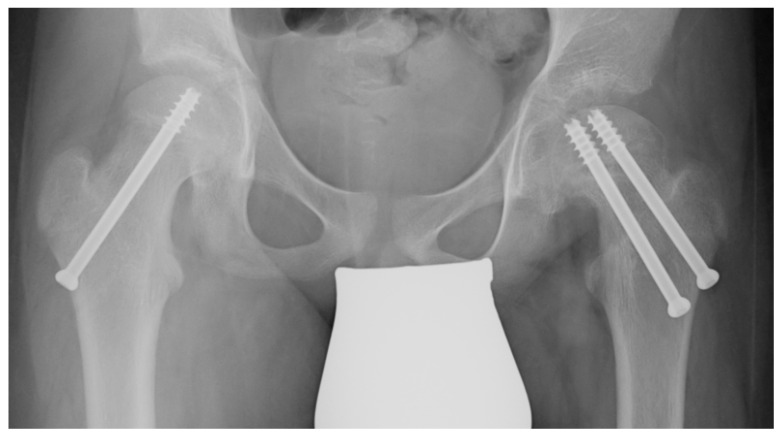
A patient treated in the left hip using VASSCO and stabilized with two screws. On the right, a single cannulated screw is placed to reduce the risk of metachronous slippage.

When the resection of the hinge is completed, the osteotomy is closed, reducing the head over the neck using a reducing maneuver. To check the correct alignment, a C-arm intensifier is used. Since the osteotomy slightly shortens the proximal femur, the reduction maneuver does not stretch the retinacular vessels. Making a lateral incision, under a fluoroscopic guide, two cannulated screws are used to fix the osteotomy. The joint capsule is carefully closed, and then the wounds are closed in layers. Prophylactic stabilization of the contralateral hip is usually performed using a single cannulated screw to reduce the risk of metachronous slippage (Figure 2) [23].

### 2.3. Postoperative Care

In the postoperative period, the hip is blocked in a spica cast for 4 weeks to control pain and to reduce the risk of screw cut-off. Although a cast could be not performed in some cases, one month without weight-bearing is mandatory.

At four weeks, the cast is removed, anteroposterior and frog-leg lateral X-ray views are taken of both hips, partial weight-bearing is mandated for six more weeks, and exercises to regain the full hip range of motion are prescribed. At 10 weeks from surgery, full weight-bearing is permitted as tolerated.

### 2.4. Clinical and Radiological Outcomes

**Figure 3:**
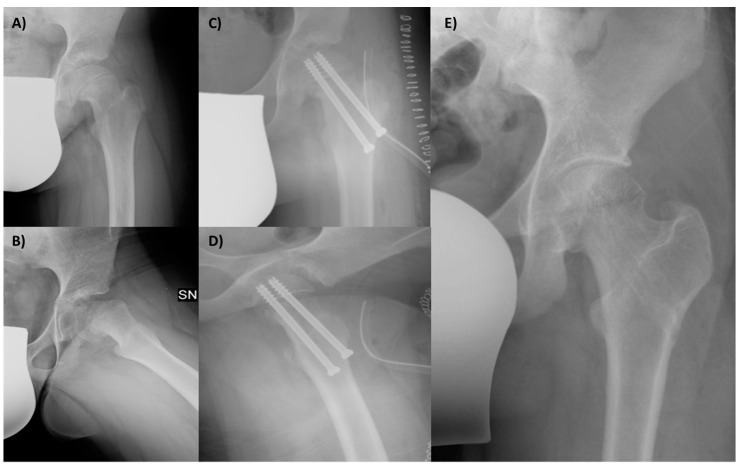
A 12-year-old male patient affected by SCFE, with great deformity of the proximal femur, with a varus (**A**) and retroverted (**B**) neck. The femur after the surgery, with the correction in the coronal (**C**) and sagittal planes (**D**); the screws in both views are in the bone and not protrude into the joint. The final X-ray follow-up at 8 years shows the outcome of removing screws and the radiological good hip joint status (**E**).

To evaluate the hip function, we used the Harris Hip Score (HHS) and hip joint articulation in the pre-operative period and at 3 and 6 months and 1 and 8 years follow-up. Anteroposterior and frog-leg lateral X-ray views were taken preoperatively and then at 4 weeks, 6 months, and yearly after. The level of slippage [24] was recorded in the pre-operative period and at last follow-up (Figure 3). The lateral α angle [25] and the epiphyseal–metaphyseal offset [26] were also evaluated preoperatively and at the latest follow-up.

**Figure 4:**
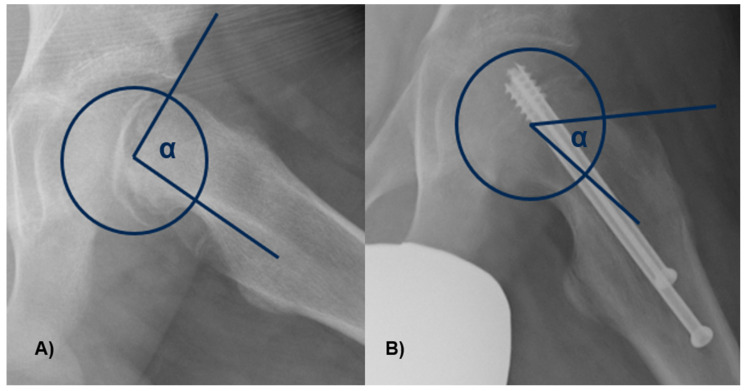
The α angle was measured between the axis of the femoral neck and the line from the center of the femoral head to the point where the distance from the center of the femoral head to the peripheral contour of the femoral head exceeded the radius of the femoral head. In figure (**A**), there is an angle greater than 90°, with neck deformity, while in figure (**B**), the angle and the deformity are lessened.

The α angle was measured between the axis of the femoral neck and the line extending from the central part of the femoral head to the location where the distance from the central point of the femoral head to the outer edge surpassed the femoral head’s radius (Figure 4). Slippage of the femoral head will occur at a higher α angle.

**Figure 5:**
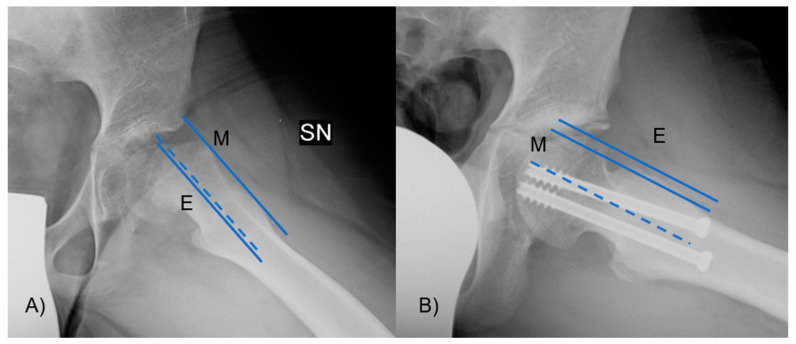
The epiphyseal–metaphyseal offset was measured in the frog-leg lateral view as the difference between (E) a line drawn along the anterior edge of the epiphysis, parallel to the femoral neck, and (M) a line drawn along the proximal–anterior edge of the metaphysis, also parallel to the femoral neck. In figure (**A**) the offset is reduced due to the head slippage, while (**B**) after the reduction it is improved.

The epiphyseal–metaphyseal offset was assessed in the frog-leg lateral view by determining the disparity between a line traced along the front border of the epiphysis, parallel to the femoral neck, and a line traced along the proximal–anterior edge of the metaphysis, also parallel to the femoral neck (Figure 5). A negative value means that the epiphysis lies posteriorly to the metaphysis. Finally, the presence of AVN or cartilage degeneration was evaluated at the latest available follow-up.

### 2.5. Statistical Analysis

Analyses were conducted comparing the groups pre- and postoperatively using Student’s t-test; significance was set at a *p*-value < 0.05. (SPSS 14.0, version 14.0.1 (SPSS Inc., Chicago, IL, USA).

## 3. Results

From a total of 62 proximal femur osteotomies performed from 2012 to April 2016, 26 patients matched the inclusion criteria and were retrieved for analysis; the patients excluded were treated with the modified Dunn procedure and subtrochanteric osteotomy. A further 2 patients were lost during follow-up, leaving 24 patients under investigation. The patients lost at clinical and radiological follow-up were phone-called to exclude AVN, and both confirmed that no further surgeries were performed on their hips. A final 24 patients were included (19 males and 5 females; 79% M and 21% F).

The average age at surgery was 12.3 (range 10–14 years), without a significant difference between males and females. The average follow-up of patients was 10 years (range 8–12 years).

**Figure 6:**
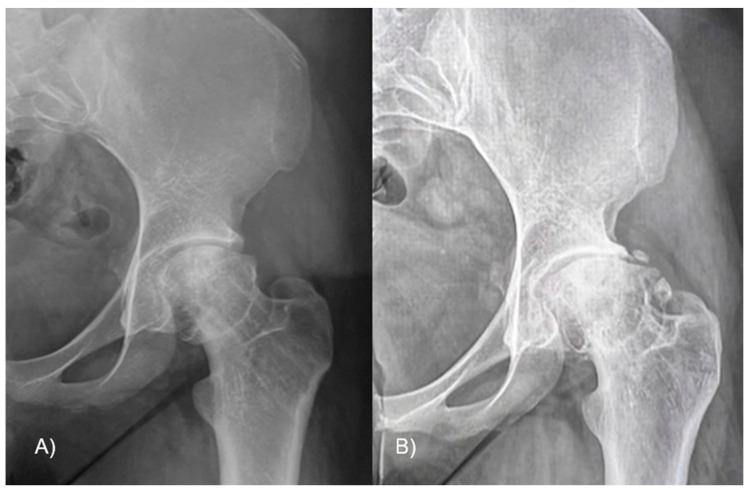
(**A**) Patient 10 months after VASSCO, after the removal of the cannulated screws. (**B**) After 12 years, secondary osteoarthritis developed, with severe limitations, which required total hip arthroplasty.

No major intraoperative complications occurred; there were three cases of postoperative transient deficits in the lateral femoral cutaneous nerve, resulting in thigh hypoesthesia. Fortunately, the hypoesthesia recovered within a year after the initial surgery. In one patient, 6 months after surgery, AVN of the femoral head occurred (1/24; 4.2% rate). This patient was treated 8 months before the index surgery for subacute SCFE reduction and percutaneous pinning. The patient was treated with the removal of the cannulated screws and no weight-bearing and crutches; in addition, physical therapy was performed. After 12 years, the patient developed secondary osteoarthritis and was treated with total hip arthroplasty (Figure 6).

In all the other patients, at the last available follow-up, there was an improvement in the hip range of motion; the FADIR test was negative in 21 out of the 24 patients. The average joint articulation was 13° of internal rotation (minus 3°–21° range) and 84° of flexion (74–105° range) preoperatively. At the latest follow-up, the hip ROM was 36° of internal rotation (30–40° range) and 114° of flexion (105–124° range) (*p* = 0.022). The preoperative HHS was, on average, 64 (range, 48–81), while at the last follow-up, it averaged 92.5 (range, 87–98) (*p* = 0.007) (Table 1).

Preoperatively, 7 out of 24 patients were evaluated as grave cases (29%) and 17 were evaluated as mild (71%); the slippage in the preoperative period was 44.3° (35–53° range). After surgery, there was an average slippage of 12.2° (range, 8–17°) (*p* = 0.033). The initial lateral α angle measured at 94° (range, 62–113°) on the radiographs before the operation, and the subsequent average postoperative angle was 47.5° (range, 42–55°) (*p* = 0.02). Preoperatively, the–metaphyseal offset averaged −3.3 mm (range, −6.2 to −0.4 mm), and postoperatively, it was 0.24 mm (range, −1.5 to 1.2 mm) (*p* = 0.01). Degenerative changes were absent in 20 hips and mild in 3 hips, and 1 hip showed significant morphological alterations at the femoral head due to AVN. 

The orthopedic synthesis devices were removed from all hips 6 to 12 months after the procedure. In a single patient, one screw was extracted a month after the surgery, as it intruded into the hip cartilage and caused acetabular impingement during physiotherapy; in two hips, the screws were removed 3 months after surgery because of pain in the tensor fasciae latae.

## 4. Discussion

The aim of this paper was to present the middle- to long-term results in a consecutive series of 24 patients operated on from April 2012 to December 2016 and followed up for 10 years after VASSCO surgery for the treatment of C-SCFE.

The nature of the surgical correction of C-SCFE and the method to use is still a matter of debate. Simple stabilization of the deformity in the case of moderate to severe deformity has inadequate results despite the potential of the proximal femur bone to be remodeled [4,27]. In 2013, Akiyama et al. [4] reported, in a multicentric study on 69 hips in 56 patients managed at three different hospitals using an in situ pinning technique, that 29.4% of treated patients presented residual cam deformity [4]. However, the chance to fully correct a deformity by acting at its apex is potentially beneficial in the long term because it could decrease the risk of early secondary degenerative arthritis developing.

The early clinical results on the VASSCO technique were reported in a previous study [14], which showed excellent results both clinically and radiologically, with no episode of AVN determined in the previous follow-up. In the current study, the clinical series was updated, including 15 more pediatric patients operated on using the same technique; all of these were followed up 10 years after. Analyzing the cohort, one case of AVN was recorded, with a rate of 4.2% of AVN. It occurred in a patient with previous in situ pinning performed 8 months prior to the VASSCO surgery. We have to consider that that previous treatment (in situ) could have represented an independent risk factor in the development of AVN; in fact, Bellemans et al. [28] reported a 10% risk of AVN with in situ pinning, raising the possibility of VASSCO not being the cause of the failure. After this case, we further evaluated all the hips previously operated on, and we informed the parents of the patients about the increased risk of AVN when surgery had been performed in the previous 12 months. All the remaining patients had very good clinical and radiographic results, which were retained over time.

These findings are in contrast with those reported in the literature, in which a higher rate of AVN is usually associated with subcapital osteotomy surgery. All the different techniques proposed for the management of patients affected by chronic SCFE have shown no clear advantage over the VASSCO technique.

Considering all the studies reporting on subcapital osteotomies with an adapted Watson-Jones approach involving trochanteric osteotomy with the dislocation of the hip, after the first study by Dunn et al. [6], the rate of AVN ranges between 12 and 17%.

To avoid this high rate of AVN, several authors, lead by Ganz, suggested a transtrochanteric approach with hip dislocation [29]. According to this technique, a trochanteric osteotomy is performed; the ligamentum teres is transected, retaining the subperiosteal pane with the vascular structures; and after that, the joint is anteriorly dislocated. The femoral head deformity is evaluated and corrected using a subcapital osteotomy, which is fixed afterward. A theoretical advantage of this technique is the very good exposure of the pathological anatomy of the femoral head allowed by the hip dislocation [9,12,13]. Otherwise, the dislocation is an AVN risk itself, and this technique reduces this type of complication, with the rate reported in the literature ranging from 0 to 20% [9,10,11,12,13,15].

Other authors reported a variable failure rate due to AVN. Valenza et al. [30] in 2022 described 20 patients treated using a modified Dunn technique, reporting a 45% complication rate, including five cases of AVN (25%). The authors commented that the learning curve for this surgery is long, and it requires training to minimize complications: in the study, they reported four cases of AVN in the first 5 years of practice, and one afterward [11,12,15,31]. Moreover, the trochanteric osteotomy weakens the gluteus medius, and this could pose a significant disadvantage, especially considering the typical youthful age of these patients and their future prospects in sports.

Instead, VASSCO allows for good exposure of the pathological part of the femoral head, avoiding hip dislocation and trochanteric osteotomy, and it is less demanding: the absence of dislocation and careful capsulotomy may be protect against the risk of AVN. The most delicate part of the surgery is the proper use of the Kerrison rongeur at the hinge to preserve the vascular supply at the periosteum.

These results are also supported by the findings of Guo et al. [31] that showed a significant reduction in AVN in patients affected by Pipkin fractures and treated using an anterior approach compared to a transtrochanteric approach [31]. However, if a standard anterior approach is used and all the abovementioned steps of the VASSCO technique are not followed, the results may be compromised. In fact, De Rosa et al. [17] reported a 15% risk of AVN in 27 hips treated for severe SCFE using subcapital osteotomy performed with a classic Smith-Petersen incision or a transverse incision. Interestingly, in two out of four patients that showed AVN, a closed reduction was made prior to surgery.

Birings et al. [32] recorded 25 SCFE cases treated using a standard anterior approach and reported a 12% risk of AVN with a follow-up of 8 years (Table 2).

These results suggest that the advantage of the anterior approach in these patients regards the preservation of the soft tissue and the possibility of exposing the pathological region of the head and neck; conversely, capsular preservation and a careful osteotomy technique are the crucial tips for VASSCO surgery. The key point is not to violate the posteromedial periosteum at the hinge because there lies the vascular bundle that supplies the femoral head. Furthermore, the cuneiform wedge resection, reducing the length of the femoral neck, reduces the risk of stretching over the posterior retinacular vessels.

When AVN occurs, it is fundamental to identify the severity of the lesion. In the case of mild or moderate necrosis, it is possible to treat the patient with no weight-bearing and using crutches, with associated intensive physiotherapy in order to maintain the joint ROM. In the case of severe damage, femoral head surgical options are available, such as lateral shelf acetabuloplasty or femoral osteotomy [33]. Birings [32,34] reported three cases of AVN (12%) in which two patients had partial segmental avascular necrosis and the third had complete involvement of the femoral head. The two patients were treated conservatively, while the patient with extensive AVN of the femoral head had their synthesis devices removed at one year, and then a remodeling acetabuloplasty was performed 18 months after the first operation, followed by a proximal femoral osteotomy 4.5 years after the initial treatment. In our experience, the only case, mild–moderate AVN, was treated in a conservative way, but at 12 years of follow-up, the patient reported severe secondary osteoarthritis, so a total hip arthroplasty was performed.

This study has some limitations. Because it is a retrospective study, it is associated with a selection bias. Moreover, the collected patients were operated on in a surgical unit with great expertise on the anterior approach, which is currently used in over 90% of THAs for both primary and revision surgeries. The study cohort is limited, with 24 patients selected. However, this is the only available middle- to long-term follow-up study, which averaged 10 years.

## 5. Conclusions

In conclusion, based on the current findings, VASSCO is considered a reliable technique to reduce the femoral head deformity in pediatric patients affected by chronic SFCE; the risk of AVN is extremely reduced compared to in other reports in the literature, suggesting the positive impact of the technique on the reported outcomes.

## Figures and Tables

**Table 1:** Radiographic evaluation of affected hips pre- and postoperatively.

Timing	Southwick Angle	Lateral α Angle	Epiphyseal–Metaphyseal Offset (mm)
Preop	44° ± 6.3°	94° ± 15°	−2.9 ± 2
Postop	12° ± 2.8°	47° ± 3.8°	0.2 ± 0.6

**Table 2:** Summarized studies with subcapital osteotomy.

	Patients	Follow-Up	AVN
Valenza et al. [30]	20	5 years	25%
De Rosa et al. [17]	27	8 years	15%
Birings et al. [32]	25	8 years	12%

## Data Availability

All collected data are reported in the current manuscript.
